# Biological Activity and Safety of *Tripterygium* Extract Prepared by Sodium Carbonate Extraction

**DOI:** 10.3390/molecules170911113

**Published:** 2012-09-17

**Authors:** Wei Fang, Fan Peng, Tao Yi, Cong Zhang, Chunxi Wan, Huibi Xu, Christopher Waikei Lam, Xiangliang Yang

**Affiliations:** 1 College of Life Science and Technology, Huazhong University of Science and Technology, Wuhan 430074, China; 2 State Key Laboratory for Quality Research in Chinese Medicine, Macau Institute for Applied Research in Medicine and Health, Macau University of Science and Technology, Macau, China

**Keywords:** *Tripterygium wilfordii* Hook. F., active components, sodium carbonate extraction, toxicity

## Abstract

The commercial preparation named “*Tripterygium* glycosides” prepared by column chromatography has been used for the treatment of inflammatory and autoimmune diseases with significant efficacy but concurrent toxicity. The aim of this study was to reduce the toxicity of *Tripterygium* extracts, using cytotoxicity and anti-inflammatory activity of the three principal active components of *Tripterygium wilfordii* Hook. F. (TWHF) as guiding parameters. Column chromatography was replaced by sodium carbonate extraction for removing the acidic compounds and enriching epoxyditerpenoids and alkaloids in the extract. Results showed that the therapeutic index (IC_50_/EC_50_) on murine macrophage Raw 264.7 cells and rat mesangial HBZY-1 cells of the extract prepared by sodium carbonate extraction was significantly higher than that of *Tripterygium* glycosides (0.8 and 5.2 *vs.* 0.3 and 2.6, *p* < 0.05), while its cytotoxicity on human liver HL7702 cells was significantly lower (14.5 ± 1.4 *vs.* 6.8 ± 0.9, *p* < 0.05). Further acute oral toxicity experiments showed that the LD_50_ value of this extract was 1,210 mg/kg compared to 257 mg/kg for *Tripterygium* glycosides. All the above results suggest that *Tripterygium* extract prepared by sodium carbonate extraction may represent a potentially optimal source of medicine with good therapeutic index.

## 1. Introduction

*Tripterygium wilfordii* Hook. F. (TWHF) is a woody vine widely distributed in eastern and southern China. Known in Chinese language as “Lei Gong Teng” (Thunder God Vine), this plant has a long history of therapeutic use in traditional Chinese Medicine (TCM), with efficacies in expelling wind, removing dampness, promoting blood circulation, freeing channels, trimming bruises, and as a hemagogue [1]. Commercial preparations of TWHF have been commonly used for the treatment of inflammatory and autoimmune diseases, such as rheumatoid arthritis, systemic lupus erythematosus, nephritis and psoriasis [2,3,4,5].

TWHF fractions for research and clinical studies have been extracted using different solvents: water, ethyl acetate, ethanol, and chloroform-alcohol, consequently, these extracts have different compositions and biological activities. However, extracts also manifested recurrent toxicity symptoms, such as diarrhea, leucopenia, thrombocytopenia, skin pigmentation, liver and renal injuries, and infertility [6]. Column chromatography of the ethyl acetate extract yields *Tripterygium* glycosides of reduced toxicity [7]. Wilforlide A, one of the triterpenoids, has been chosen as the index for assessing *Tripterygium* glycoside preparations. The content of this compound in TWHF is however minute (<0.1 mg/g). On the other hand, it has been generally recognized that triptolide, celastrol and wilforine are representative compounds in TWHF. Their contents in *Tripterygium* glycosides have not been investigated and the relationship of their therapeutic efficacy and toxicity has remained unclear. We studied the cytotoxicity and anti-inflammatory activity of these three principal terpenoids as the target compounds.

In addition, column chromatography is a tedious fractionation procedure with poor reproducibility affecting the yield of active compounds [8]. Guided by assessment of cytotoxicity and anti-inflammatory activity of the active parts, our objective was also to develop a simple and reproducible method for extraction of *Tripterygium* for simultaneously optimizing biological activity and reducing toxicity.

## 2. Results and Discussion

### 2.1. Cytotoxicity and Anti-inflammatory Activity of Triptolide, Celastrol and Wilforine

Toxicity and anti-inflammatory activity of the compounds were assessed initially by their IC_50_ (dosage causing 50% cell death) and EC_50_ (dosage inhibiting 50% NO and TNF-α production) values. Since the active components of TWHF always possess toxicity, the ratio of cytotoxicity to bioactivity (IC_50_/EC_50_) was used for further assessing safety and therapeutic efficacy.

As shown in Table 1, triptolide exhibited a larger therapeutic window than celastrol on murine mesangial HBZY-1 cells (IC_50_/EC_50_ > 10). This suggests that triptolide may be more efficacious and safer than celastrol for treating nephritis. Wilforine showed no obvious activity under the experimental conditions on both renal and immune cells. We consider alkaloids having moderate biological activity but can reconcile the toxic effect of the diterpenes, making the combination safer. The alkaloid fraction should therefore be retained.

**Table 1:** Cytotoxicity and anti-inflammatory activity of triptolide, celastrol and wilforine.

Sample	HBZY-1 cells		IC_50_/EC_50_	RAW264.7 cells		IC_50_/EC_50_
	Cytotoxicity IC_50_ (nmol/L)	Anti-inflammatory activity EC_50_ (nmol/L)		Cytotoxicity IC_50_ (nmol/L)	Anti-inflammatory activity EC_50_ (nmol/L)	
Triptolide	230.3 ± 9.9	20.7 ± 2.1	11.1	40.2 ± 2.7	25.0 ± 4.4	1.6
Celastrol	3715 ± 342	1040 ± 40	3.6	360 ± 41	260.3 ± 20.9	1.4
Wilforine	nd	nd	nd	nd	nd	nd

nd = not detected.

### 2.2. Quantification of Active Compounds in TWHF Extract

Many terpenoids with strong biological activities have been isolated from TWHF extracts. They comprise sesquiterpenes, diterpenes and triterpenes [9]. Celastrol, a quinonemethide triterpene, triptolide, a diterpenoid triepoxide, and wilforine, a sesquiterpene alkaloid, are representative compounds [9,10,11]. Therefore the contents of celastrol, triptolide and wilforine were measured to study the variability of active components in different extracts.

In order to reduce toxicity, the ethyl acetate extract was further extracted with sodium carbonate solution for removing the acidic components, in particular the triterpenoid celastrol, and enriching alkaloids and diterpenoids which are the two principal groups of active components of TWHF. Again, the rationale of this further extraction was that firstly, the liver toxicity of the alkaloids is much lower than those of the diterpenoids and secondly, celastrol is more nephrotxic and can cause glomerulo-nephritis and -proteinuria.

The weights and contents of celastrol, triptolide, and wilforine in the various extracts are shown in Table 2. Compared to the ethanol extract, contents of these three compounds increased approximately 2-fold in the EtOAc extract (1.4 *vs.* 0.71, 0.032 *vs.* 0.015, and 2.4 *vs.* 1.1%), and extraction yields were 89, 98.8, 100.1%, respectively. Ethyl acetate may therefore be a good solvent for extracting active compounds from the ethanol extract. In the extraction with aqueous sodium carbonate solution, the acidic component celastrol was distributed mainly in the alkaline aqueous layer at 1.7 times higher levels than the EtOAc extract (2.4 *vs.* 1.4%), while the neutral component triptolide and the weakly basic component wilforine were concentrated in the ethyl acetate layer, where their contents were 1.6 and 1.8 times higher than those in the EtOAc extract (0.051 *vs.* 0.032 and 4.4 *vs.* 2.4%), respectively.

**Table 2:** Weights and contents of celastrol, triptolide and wilforine in different extracts.

Sample	Content (%) of active compound			Weight (mg)
	triptolide	celastrol	wilforine	
Ethanol extract	0.015	0.7	1.1	1,000.0
EtOAc extract	0.032	1.4	2.4	454.5 **
Extract 1	0.051	0.09	4.4	234.0 **
Extract 2	nd *	2.4	nd	184.6 **
*Tripterygium* glycosides	0.92	nd *	1.7	-

* not detected; ** Based on the starting weight of ethanol extract at 1,000.0 mg.

### 2.3. Cytotoxicity and Anti-Inflammatory Activity of TWHF Extracts

Table 3 shows the cytotoxic activities of different extracts as assessed by the MTT assay. They provide information about the initial toxicity of extracts on different cell lines. It can be seen that IC_50_ values of the EtOAc extract were the lowest among all extracts, confirming that this extract had the highest toxicicty. With sodium carbonate extraction, toxicity was greatly reduced, especially on human HL7702 liver cells (*p* < 0.05). Compared to the EtOAc extract, the toxicity of Extract 1 (containing mainly neutral and weakly basic compounds) had reduced about three times (4.9 to 14.5), and that of Extract 2 (containing mainly acidic components) about eight times (4.9 to 33.3) on HL7702 cells. The concentrations of active compounds in EtOAc extract were less than those in Extract 1 (0.032 *vs.* 0.051 and 2.4 *vs.* 4.4% for triptolide and wilforine) and Extract 2 (1.4 *vs.* 2.4% for celastrol). Toxicity may therefore be influenced by the interaction of multiple components in TWHF. Removal of some compounds may reduce the side effects of *Tripterygium* preparations.

**Table 3:** Cytotoxic and anti-inflammatory activities of different extracts.

Sample	HBZY-1 cells			RAW264.7 cells			HL7702 cells
	Cytotoxicity IC_50_ (μg/mL)	Anti-inflammatory activity EC_50_ (μg/mL)	IC_50_/EC_50_	Cytotoxicity IC_50_ (μg/mL)	Anti-inflammatory activity EC_50_ (μg/mL)	IC_50_/EC_50_	Cytotoxicity IC_50_ (μg/mL)
EtOAc Extract	7.3 ± 1.1	1.3 ± 0.2	5.6	1.2 ± 0.3	2.3 ± 0.9	0.5	4.9 ± 0.3
Extract 1	12.5 ± 2.0 *	2.4 ± 0.3 *	5.2	5.3 ± 0.4 *^,#^	6.6 ± 0.3 *^,#^	0.8	14.5 ± 1.4 *^,#^
Extract 2	11.7 ± 1.8 *	2.7 ± 0.6 *	4.3	1.7 ± 0.2	2.0 ± 1.0	0.85	33.3 ± 0.2 *
*Tripterygium* glycosides	4.0 ± 0.2	1.5 ± 0.3	2.6	1.7 ± 0.5	5.3 ± 0.6 *	0.3	6.8 ± 0.9

Compared to EtOAc extract: * *p* < 0.05, Compared to Extract 2: ^#^*p* < 0.05.

Compared to the EtOAc extract, toxicity of Extract 1 on RAW264.7 cells was reduced about four times (1.2 to 5.3, *p* < 0.05), and that of Extract 2 was comparable (1.2 *vs.* 1.7). The content of celastrol in Extract 1 was lower than those of the EtOAc extract and Extract 2 (0.09 *vs.* 1.4 and 2.4). Therefore celastrol should be viewed as the principal toxic compound in TWHF that was removed for reducing the toxicity of TWHF extracts [12].

All extracts showed significantly dose-dependent anti-inflammatory activity, as assessed by their *in vitro* inhibition of LPS-induced TNF-α from macrophages and NO production from rat mesangial cells. As shown in Table 3, EC_50_ values of the EtOAc extract was lower than those of Extract 1 and Extract 2. This demonstrated that the EtOAc extract was biologically the most active one. Such a finding is in agreement with the cytotoxic assessment, which can be explained by the presence of compounds, such as diterpenoid lactone epoxides [13] and celastrol [14], not only having significantly anti-inflammatory and immunosuppressive activity, but also severe toxicity. The biological activity of TWHF extracts is related to its toxicity, it is important to balance the two effects therapeutically [15].

With regard to the contents of active compounds in *Tripterygium* glycosides and Extracts 1, Table 2 shows that the contents of wilforine and triptolide in Extract 1 were 4.4 and 0.051%, respectively, while those in *Tripterygium* glycosides were 1.70, and 0.92%, respectively. Wilforine in Extract 1 was therefore 2.6 times more abundant than that in *Tripterygium* glycosides, while the content of triptolide was only an 1/18 fraction. Even with such large differences between the contents of the two active compounds, the anti-inflammatory activities of Extract 1 and *Tripterygium* glycosides were comparable, and the therapeutic index (IC_50_/EC_50_) of Extract 1 was higher than that of *Tripterygium* glycosides on Raw 264.7 and HBZY-1 cells. Furthermore, the composition of Extract 1 (containing more alkaloids) suggested that its toxicity should be lower than that of *Tripterygium* glycosides.

### 2.4. Assessment of Extract 1 for Acute Toxicity

The experimental animals showed obvious toxicity from oral administration of Extract 1 manifesting slow movements, diarrhea and respiratory depression. The high-dose group died within 24 h, and all dead mice showed significant gastrointestinal toxicity, intestinal necrosis, black liver and spleen, with toxicity being more severe in male than female mice. The LD_50_ of Extract 1 was 1,210 mg/kg, and the 95% confidence interval (CI) was 1,098–1,322 mg/kg. As reported in the literature, LD_50_ of the *Tripterygium* glycosides tablet is 257 mg/kg (CI = 227 − 287 mg/kg) [16]. Thus our study showed that with sodium carbonate extraction, the toxicity of *Tripterygium* extract was greatly reduced.

## 3. Experimental

### 3.1. Materials

All solvents were of HPLC grade, purchased from Merck Serono Co. Ltd. (Darmstadt, Germany). Triptolide was purchased from the National Institute for Food and Drug Control (Beijing, China), and celastrol from Zelang Pharmaceutical Technology Co. Ltd. (Nanjing, China). Wilforine was isolated and identified by our laboratory. The purity of these three terpenoids was more than 98% as determined by high performance liquid chromatography (HPLC).

RPMI-1640, DMEM/high glucose, fetal bovine serum (FBS), streptomycin sulphate and ampicillin, trypsin, ethylenediamine tetraacetic acid (EDTA), L-glutamate and non essential amino acid were purchased from Gibco (Invitrogen Co., Burlington, ON, Canada), Lipopolysaccharide (LPS; *Samonella minnesota*), dimethyl sulfoxide (DMSO), 3-(4,5-dimethyl-2-thiazolyl)-2,5-diphenyl-2H-tetrazolium bromide (MTT), sodium lauryl sulfonate (SDS) and Griess reagent were purchased from Sigma-Aldrich Co. (St. Louis, MO, USA). Dimethylformamide (DMF), *N*-(1-naphthyl)-ethylenediamine dihydrochloride and 4-aminobenzenesulfonic acid were purchased from Sinopharm Chemical Reagent Co. Ltd. (Shanghai, China). Recombinant rat TNF-α, purified anti-rat/mouse TNF-α (clone TN3-9,10Ab), biotin-conjugated anti-rat/mouse TNF-α (poly clone, 20Ab), Super AquaBlue ELISA substrate and Avidin-HRP were purchased from eBioscience Co. (San Diego, CA, USA).

### 3.2. Assessment of Cytotoxicity Using MTT Assay for Triptolide, Celastrol and Wilforine

#### 3.2.1. Cell Culture and Treatment

The human liver HL-7702 cell line (ATCC CRL-11731) and murine macrophage-like cell line RAW 264.7 (ATCC TIB-71), both purchased from Wuhan Boster Bio-engineer Co. Ltd. (Wuhan, China), were maintained in RPMI-1640 medium supplemented with 10% (v/v) FBS, 1% (w/v) L-glutamate, 1% (w/v) non-essential amino acid, 0.01% (w/v) ampicillin and 0.05% (w/v) streptomycin sulphate (RPMI-1640 complete medium). The rat mesangial cell line HBZY-1 (CCTCC GDC-124), purchased from China Center for Type Culture Collection (Wuhan, China), was maintained in Dulbecco’s modified Eagle’s medium (DMEM) supplemented with 10% (v/v) FBS, 1% (w/v) L-glutamate, 1% (w/v) non essential amino acid, 0.01% (w/v) ampicillin and 0.05% (w/v) streptomycin sulphate (DMEM complete medium). All cells were incubated at 37 °C in air atmosphere enriched with 5% (v/v) CO_2_. HL-7702 cells were subsequently subcultured using 0.25% (w/v) trypsin-1mM EDTA with gentle aspiration to form a single cell suspension, while RAW 264.7 cells were subcultured using cellular scraper to form a single cell suspension. At 80% confluence, cells dispersed in RPMI-1640 or DMEM complete medium to a final cell concentration of 2 × 10^5^ cells/mL.

#### 3.2.2. MTT Assay

Assessment of cytotoxic activity was based on the reduction of MTT by the mitochondrial dehydrogenase of viable cells to give a blue formazan product, which can be measured spectrophotometrically. Briefly, cells were seeded in a 96-well plate at density 5 × 10^3^ cells per well with 180 μL complete RPMI medium and incubated for 12 h at 37 °C for adherence. 20 μL of samples (dissolved in DMSO at various concentrations), blank control (water) and negative control (2% (w/v) DMSO) were mixed into the well, and cells were incubated at 37 °C for 72 h. MTT solution (5 mg/mL) was added to each well at 5 μL/well), and the plate was incubated for another 12 h. The supernatant was carefully removed and replaced with 100 μL of cell lysis buffer [40% (w/v) SDS, 50% (w/v) DMF and 50% water, pH = 4.7], and the plate agitated to dissolve the formazan crystals. The absorbance of the dissolved formazan product was measured at 492 nm in a microplate reader. Cell viability was calculated using the following formula: % viability = absorbance of sample/absorbance of control × 100.

### 3.3. Anti-inflammatory Assays for Triptolide, Celastrol and Wilforine

#### 3.3.1. Measurement of Nitrite

HBZY-1 cells were seeded into a 48-well culture plate at density of 5 × 10^4^ cells per well with 400 μL of DMEM complete medium, and incubated for 12 h. The complete medium was replaced with 360 μL serum-free DMEM medium, 20 μL of sample (dissolved in DMSO at various concentrations), blank control (water) and negative control (2% w/v DMSO). Cells were then stimulated with 20 μL LPS (100 ng/mL) for 48 h. The presence of nitrite, a stable oxidized product of nitric oxide (NO) from anti-inflammatory activity of the cells against LPS, was determined in cell culture medium using Griess reagent. In this measurement, 50 μL supernatant was removed and reacted with 50 μL Griess reagent in a 96-well plate, followed by spectrophotometric measurement at 531 nm on a microplate reader. The cell viability was determined using MTT assay.

#### 3.3.2. ELISA

RAW 264.7 cells were seeded into a 48-well culture plate at 5 × 10^4^ cells per well with 360 μL of RPMI-1640 complete medium, and incubated for 12 h. 20 μL of sample (dissolved in DMSO at various concentrations), blank control (water) or negative control (2% w/v DMSO) was added into the well, and cells were stimulated with 20 μL LPS (20 ng/mL). The supernatant of each well was collected after 12 h and stored at −20 °C.

Double-antibody “sandwich” ELISA was used to determine TNF-α concentration in the samples. Briefly, Nunc MaxiSorp flat-bottom 96-well plate (eBioscience) was coated with primary antibody at 4 °C overnight, and the plate was blocked with blocking buffer (1% BSA, 5% sucrose, 0.05% NaN_3_ in PBS (pH = 7.2–7.4, Ca^2+^ and Mg^2+^ free) at room temperature for at least 2 h. After washing 5 times with wash buffer (PBS with 0.05% Tween-20), samples were added into the appropriate wells, and the plate was incubated at 4 °C overnight. Secondary antibody (diluted with Assay Buffer (PBS with 0.05% Tween-20 and 0.1% BSA) was added into each well after the plate was washed with wash buffer. After 1 h at room temperature, the plate was washed again, Avidin-horse reddish peroxidase (HRP) solution was added to all the wells, and protected from light at room temperature for 1 h. After washing, Super AquaBlue ELISA substrate Solution was added to each well. The plate was incubated at room temperature for 30 min, and read at 405 nm.

### 3.4. Preparation of Plant Extracts

*T. wilfordii* roots were collected from a Good Agricultural Practice (GAP) site in Taining County of Fujian province in January 2010 and identified by Professor Xiaochuan Ye of Hubei University of Chinese Medicine. Voucher specimens were deposited in Institute of Materia Medica of Huazhong University of Science and Technology with reference numbers THWF 1001151.

**Figure 1:**
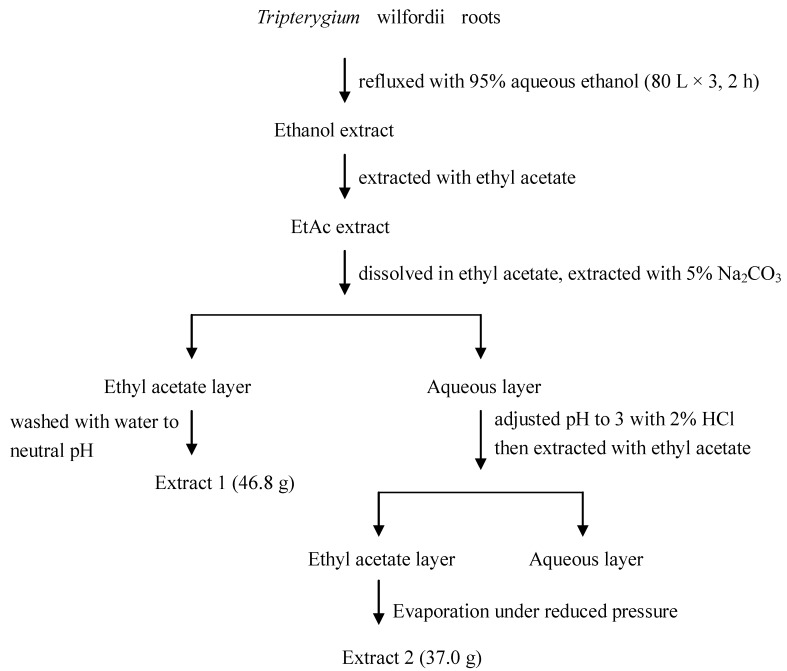
Protocol for preparation of different TWHF extracts.

Roots (16 kg) were cut into small pieces and dried at 50 °C, then ground into 40–60 mesh powder. The powder was extracted three times by refluxing with 95% aqueous ethanol (80 L) for 2 h. The ethanol solvent was filtered and dried by evaporation under reduced pressure to yield the Ethanol extract (380 g). The Ethanol extract (200 g) were extracted three times by refluxing with ethyl acetate (2 L) for 1 h. The ethyl acetate solvent was then filtered and evaporated under reduced pressure to yield the EtOAc extract. This extract (90 g) was dissolved in ethyl acetate, extracted with 5% sodium carbonate solution twice, and then washed with water to neutral pH, and evaporated under reduced pressure to yield Extract 1. Sodium carbonate solution and washing water were combined, the pH of the mixture was adjusted to 3 with 2% hydrochloric acid, and extracted three times with ethyl acetate. The ethyl acetate layer was evaporated under reduced pressure to yield Extract 2 (Figure 1). The *Tripterygium* glycoside medicine (100 tablets, Lot No.20100302) was ground into power and extracted three times by refluxing with methanol (100 mL) for 1 h. The methanol solvent was filtered and then dried by evaporation under reduced pressure to yield *Tripterygium* glycoside extract.

### 3.5. Measurement of Triptolide, Celastrol and Wilforine in the Extracts

#### 3.5.1. Triptolide

Triptolide content of the crude extract was determined by HPLC, as described previously [17], except with some slight modifications. The extract (0.2 g) was dissolved in chloroform (5 mL) and loaded on a column packed with 2.0 g neutral aluminum oxide (Al_2_O_3_). Elution was with ethyl acetate (20 mL). The eluate was evaporated under reduced pressure, and redissolved in methanol (5.0 mL). Samples were filtered through a 0.22 μm membrane filter for HPLC analysis, which was carried out on an Agilent 1100 HPLC system equipped with an Agilent G1314A UV detector (Agilent Technologies, Waldbronn, Germany) and Agilent Software. The separation was performed at 25 °C using a 250 mm × 4.6 mm, i.d., 5 μm, ZORBAX Eclipse Plus C18 with a Agilent guard cartridge. The mobile phase was composed of MeOH-H_2_O at 42:58 (v/v). The injection volume was 10 μL, and detection was carried out at 210 nm. In order to quantify the amount of triptolide in extracts, calibration curves were prepared with the standards dissolved in methanol. The calibration curves showed good linearity over 2.6–52.0 μg/mL.

#### 3.5.2. Celastrol

Celastrol content of the crude extract were determined by HPLC, as described previously with some modifications [18]. Extract (0.1 g) was dissolved in chloroform (5 mL) and loaded on a column packed with 2.0 g silica, Elution was with ethyl acetate (20 mL). The eluate were evaporated under reduced pressure, and re-dissolved in methanol (10.0 mL). Samples were filtered through a 0.22 μm membrane filter for HPLC, which was carried out on the same instrument with the same column as described above. The injection volume was 10 μL, and detection was carried out at 425 nm. In order to quantify the amount of celastrol in extracts, calibration curves were prepared with the standards dissolved in methanol. The calibration curves showed good linearity from 10.0 to 200.0 μg/mL

#### 3.5.3. Wilforine

Wilforine content of the crude extract was determined by HPLC, as described previously with some modifications [19]. Extract (50 mg) was dissolved in chloroform (5 mL) and loaded on a column packed with neutral Al_2_O_3_ (2.0 g). Elution was with ethyl acetate (20 mL). The eluate was evaporated under reduced pressure, and re-dissolved in methanol (5.0 mL). Samples were filtered through a 0.22 μm membrane filter for HPLC. The HPLC analysis was carried out on the same instruments with the same column as described above. The injection volume was 10 μL, and detection was carried out at 210 nm. In order to quantify the amount of wilforine in extracts, calibration curves were prepared with the standards dissolved in methanol. The calibration curves showed good linearity over 5.1–68.0 μg/mL range.

### 3.6. Cytotoxicity and Anti-inflammatory Activity of the Extracts

The extracts were assessed for cytotoxicity and antiinflammatory activity as described above.

### 3.7. Assessment of Acute Toxicity

We first assessed the range of doses of Extracts 1 that caused 0% and 100% deaths in mice using a pre-test. Fifty BALB/c mice were randomly divided into five groups (five female mice and five male mice each). Each group was administered orally different doses of Extract 1 at 725.8, 1,209.6, 2,016, 3,360 and 5,600 mg/kg body weight. After administration, all external morphological, behavioral, neurological and autonomic changes, number of deaths and time to death, as well as some other toxic effects were recorded continuously at 1 h intervals over a period of 24 h. According to the mortality of mice observed within one week, the LD_50_ values (95% confidence interval) for Extract 1 was calculated using BSAS110 software (National Institute for the Control of Pharmaceutical and Biological Products, Beijing, China) with the Bliss method.

### 3.8. Statistical Analysis

Experimental data were expressed as means ± SD. Results were analyzed by an analysis of variance (ANOVA) (*p* < 0.05) and means separated by Duncan’s test. Statistical analysis was performed using SAS program 8.2 software (SAS Institute Inc., Cary, NC, USA).

## 4. Conclusions

The results of this study showed that removal of the acidic components of *Tripterygium* using sodium carbonate extraction greatly reduced its cytotoxicity. Further acute toxicity tests confirmed that Extract 1 had the lowest toxicity and therefore could represent a potential source of raw medicine material for clinical application.

## References

[1] The Editorial Board of Chinese Materia Medica of State Administration of Traditional Chinese medicine of P. R. China. (1999). Chinese Materia Medica.

[2] Tao X., Lipsky P.E. (2000). The Chinese anti-inflammatory and immunosuppressive herbal remedy *Tripterygium wilfordii* Hook.f. Rheum. Dis. Clin. N. Am..

[3] Qiu D., Peter N.K.  (2003). Immunosuppressive and anti-Inflammatory mechanisms of triptolide, the principal active diterpenoid from the chinese medicinal herb *Tripterygium wilfordii* Hook. F. Drugs R&D.

[4] Li L.S., Liu Z.H. (2003). Experience of triptolide treatment of nephritis in twenty-five years. Chin. J. Nephrol. Dial. Transplant..

[5] Zheng C.X., Chen Z.H., Zeng C.H., Qin W.S., Li L.S., Liu Z.H. (2008). Triptolide protects podocytes from puromycin aminonucleoside induced injury *in vivo* and *in vitro*. Kidney Int..

[6] Sun X., Zhang S.M., Tian C.H., Yang L., Wang L.M., Li S.L. (2001). Safety of *Tripterygium wilfordii*. Chin. J. New Drugs.

[7] Zhang Q.P., Song H.T. (2009). The study on Preparations of TWHF. China Pharm..

[8] Gao X., Song M., Zhuang Y., Zhai Q.B., Hang T.J., Ma P.C. (2009). Study of *Tripterygium wilfordii* Hook. F. and *Tripterygium wilfordii* preparations by HPLC specific chromatograms. Chin. J. Pharm. Anal..

[9] Brinker A.M., Ma J., Lipsky P.E., Raskin I. (2007). Medicinal chemistry and pharmacology of genus *Tripterygium* (Celastraceae). Phytochemistry.

[10] Salminen A., Lehtonen M., Paimel T., Kaarniranta K. (2010). Celastrol: Molecular targets of Thunder God Vine. Biochem. Bioph. Res. Commun..

[11] Liu Q.Y. (2011). Triptolide and its expanding multiple pharmacological functions. Int. Immunopharmacol..

[12] Li H.R., Li S.F., Duan H.Q., Zhou W., Tian S.J. (2007). Process optimization of supercritical fluid extraction of active components in *Tripterygium Wilfordii* Hook.f. J. Tianjin Univ..

[13] Zheng J.R., Gu K.X., Xu L.F., Gao W.J., Yu Y.H., Tang M.Y.  (1991). Screening of active anti-inflammatory, immunosuppressive and antifertility components of Tripterygium wilfordii. III. A comparison of the anti-inflammatory and immunosuppressive activities of 7 diterpene lactone epoxide compounds *in vivo*. Acta Acad. Med. Sin..

[14] Zhu X.M., Xiong R.C., Zhang Y.F., Zhang Y.K. (1996). Studies on immunosuppresive effect of tripterine monomer on renal transplant mongrel dogs. Chin. J. Organ Transplant..

[15] Zhou J.L., Zhu Q., Yang X.L., Zhang F. (1999). Clinical observation of side effects of *Tripterygium* preparation. Chin. J. Integr. Med..

[16] Li L.Y., Jin R.M., Li Y.K., Fu S.G., Huang J., Zhu Z.L., Zhang J.H. (2006). Study immunosuppressive effect and safety *Tripterygium* glycosides by multiple doses. Chin. J. New Drugs Clin. Rem..

[17] Chi Y.M., Wen H.M., Xu J.G. (2001). Determination of content of triptolide in *Tripterygium wilfordii* herbal pieces with HPLC method. J. Nanjing Univ. Tradit. Chin. Med. Nat. Sci..

[18] Ku E., Zhang Y. (2008). Determination of content of celastrol in *Tripterygium wilfordii* herbal pieces with HPLC method. Northwest Pharmaceut. J..

[19] Huang W.H., Zhang R., Guo B.L., Si J.P. (2008). Study on total alkaloids content and its influential factors in medicinal materials of *Tripterygium*. China J. Chin. Materia Med..

